# Do interleukin-28B single nucleotide polymorphisms influence the natural history of chronic hepatitis B?

**DOI:** 10.1186/1758-2652-13-S4-P205

**Published:** 2010-11-08

**Authors:** L Martín-Carbonero, NI Rallón, JM Benito, E Poveda, J González-Lahoz, V Soriano

**Affiliations:** 1 Hospital Carlos III, Madrid, Spain

## Background

Single nucleotide polymorphisms (SNPs) nearby the IL28B gene have been associated with spontaneous hepatitis C virus (HCV) clearance and response to interferon-based therapies in both HCV-monoinfected and HIV-HCV co-infected patients. However, little is known about the impact of IL28B SNPs on HBV natural history.

## Methods

A case control study was performed in which cases were HIV+ patients with chronic hepatitis B (HBsAg+ for >6 months). All were genotyped for the rs12979860 SNP (protective CC genotype). One control for each case was chosen among HIV patients with anti-HBs and anti-HBc. Controls were matched for gender and coinfection with HCV.

## Results

A total of 49 cases fit the inclusion criteria. Most were male (90%), with a median (IQR) age of 42.6 (39-46.7) years-old. Eighteen (36.7%) were or had been chronic infected by HCV. Among HBsAg+ patients, 19 (41.3%) were HBeAg+ and 13 (26.5%) were superinfected by the hepatitis delta virus (HDV). No differences were found in the distribution of CC genotypes when comparing patients with chronic hepatitis B and those who spontaneously cleared HBsAg (59.2% vs 44.9%, respectively; p=0.3).

## Conclusions

There is no evidence for a beneficial role of the IL-28B CC genotype on the development of chronic hepatitis B in HIV-coinfected patients.

